# Backup Expression of the PhaP2 Phasin Compensates for *phaP1* Deletion in *Herbaspirillum seropedicae*, Maintaining Fitness and PHB Accumulation

**DOI:** 10.3389/fmicb.2016.00739

**Published:** 2016-05-20

**Authors:** Luis P. S. Alves, Cícero S. Teixeira, Evandro F. Tirapelle, Lucélia Donatti, Michelle Z. Tadra-Sfeir, Maria B. R. Steffens, Emanuel M. de Souza, Fabio de Oliveira Pedrosa, Leda S. Chubatsu, Marcelo Müller-Santos

**Affiliations:** ^1^Nitrogen Fixation Laboratory, Department of Biochemistry and Molecular Biology, Federal University of ParanáCuritiba, Brazil; ^2^Functional Morphology and Comparative Ecophysiology Laboratory, Cell Biology Department, Federal University of ParanáCuritiba, Brazil

**Keywords:** polyhydroxybutyrate, PHB, phasin, *Herbaspirillum seropedicae*, granule-associated proteins, backup regulation

## Abstract

Phasins are important proteins controlling poly-3-hydroxybutyrate (PHB) granules formation, their number into the cell and stability. The genome sequencing of the endophytic and diazotrophic bacterium *Herbaspirillum seropedicae* SmR1 revealed two homologous phasin genes. To verify the role of the phasins on PHB accumulation in the parental strain *H. seropedicae* SmR1, isogenic strains defective in the expression of *phaP1, phaP2* or both genes were obtained by gene deletion and characterized in this work. Despite of the high sequence similarity between PhaP1 and PhaP2, PhaP1 is the major phasin in *H. seropedicae*, since its deletion reduced PHB accumulation by ≈50% in comparison to the parental and Δ*phaP2*. Upon deletion of *phaP1*, the expression of *phaP2* was sixfold enhanced in the Δ*phaP1* strain. The responsive backup expression of *phaP2* partially rescued the Δ*phaP1* mutant, maintaining about 50% of the parental PHB level. The double mutant Δ*phaP1.2* did not accumulate PHB in any growth stage and showed a severe reduction of growth when glucose was the carbon source, a clear demonstration of negative impact in the fitness. The co-occurrence of *phaP1* and *phaP2* homologous in bacteria relatives of *H. seropedicae*, including other endophytes, indicates that the mechanism of phasin compensation by *phaP2* expression may be operating in other organisms, showing that PHB metabolism is a key factor to adaptation and efficiency of endophytic bacteria.

## Introduction

*Herbaspirillum seropedicae* SmR1 is a diazotrophic β-Proteobacterium that associates beneficially with economically relevant species of Gramineae (Baldani et al., 1986) and produces poly-3-hydroxybutyrate (PHB) granules as means of carbon and energy storage (Catalan et al., 2007; Kadowaki et al., 2011). Therefore, *H. seropedicae* SmR1 is an important model to study the impact of PHB metabolism on endophytic growth and adaptation. Thirteen genes probably involved in PHB metabolism were identified in the strain SmR1 (Kadowaki et al., 2011; Pedrosa et al., 2011), including four *phaC*, two *phaZ* and two *phaP* genes encoding PHA synthases, PHA depolymerases and phasins, respectively. Although *H. seropedicae* SmR1 possesses three genes encoding proteins homologous to PHA synthases (Pedrosa et al., 2011), PHB synthesis is supported by the PHA synthase expressed by *phaC1*, since its deletion abolishes PHB accumulation (Tirapelle et al., 2013). Furthermore, *phbF* (hereafter *phaR*) which encodes a transcriptional repressor protein was identified and characterized (Kadowaki et al., 2011). Probably, PHB plays important roles in nitrogen fixation and plant–bacteria interactions (Mandon et al., 1998; Kadouri et al., 2003; Wang et al., 2007; Quelas et al., 2013), but its action in *H. seropedicae* was not been fully characterized.

Poly-3-hydroxybutyrate is an aliphatic polyester member of the polyhydroxyalkanoates (PHA) family that some bacteria synthesize to store carbon and reducing equivalents (Anderson and Dawes, 1990; Madison and Huisman, 1999). In addition, the production of PHB is a hot topic in biotechnology due to its physicochemical properties very close to oil-based plastics, while PHB is readily degradable in the environment (Chen, 2009; Urtuvia et al., 2014). Therefore, PHB is a bio-sustainable alternative for synthetic plastic materials. PHB is usually produced under conditions of carbon excess and low levels of essential nutrients including nitrogen, phosphate, and oxygen (Hervas et al., 2008). At least three enzymes: 3-ketothiolase, acetoacetyl-CoA reductase and PHA synthase encoded by *phaA, phaB*, and *phaC*, respectively, are involved in its synthesis (Babel et al., 2001), which occurs by condensation of acetyl-CoA forming acetoacetyl-CoA, then reduction of acetoacetyl-CoA to 3-hydroxybutyryl-CoA (3HB-CoA) and finally polymerization of 3HB-CoA to yield PHB (Steinbuchel and Hein, 2001). When carbon/energy is required, the polymer is mobilized by PHA depolymerases encoded by *phaZ* genes (Babel et al., 2001). Polymeric PHB is stored as insoluble, intracellular granules that are coated with proteins (totaling 0.5–2% of the granule weight; Grage et al., 2009; Jendrossek, 2009). Phasins are small amphiphilic proteins attached on the surface of polyhydroxyalkanoate inclusions in Bacteria and Archaea (Neumann et al., 2008; Jendrossek, 2009; Cai et al., 2012). These proteins control the size and number of PHB granules (Wieczorek et al., 1995; Jurasek and Marchessault, 2002, 2004; Potter et al., 2004; Cho et al., 2012) and are present in all PHA producing bacteria. Although not highly conserved in terms of amino acid sequence, phasins perform similar functions in promoting granule formation and stabilization of PHA in different microbes (York et al., 2001a,b; Jurasek and Marchessault, 2002). *Ralstonia eutropha* H16, a well-studied model of PHB metabolism, contains seven phasin genes (Potter et al., 2005; Kuchta et al., 2007; Pfeiffer and Jendrossek, 2011, 2012) but it seems that PhaP1 is the major phasin affecting PHB accumulation (Potter et al., 2005). In plant-associated bacterium *Sinorhizobium meliloti* Rm1021, the genes SMc00777 and SMc02111 encode the phasins PhaP1 and PhaP2, respectively (Wang et al., 2007). The deletion of both genes resulted in a mutant defective in PHB production and plants of *Medicago truncatula* inoculated with this mutant exhibited reduced shoot dry weight. The occurrence of phasin-expressing genes in the genome of other plant-associated bacteria as *Azospirillum brasilense* Sp245, *A. lipoferum* 4B, *Azoarcus* sp. BH72 and *Pseudomonas stutzeri* A1501 (Krause et al., 2006; Yan et al., 2008; Wisniewski-Dye et al., 2011) indicates that PHB metabolism is important to bacteria during plant colonization, as previously suggested (Trainer and Charles, 2006). The genome sequencing of *H. seropedicae* SmR1 revealed two paralogous phasin genes (75% similarity, 59% identity), the products of which had been shown by proteomic analyses to be the main phasins associated to PHB granules (Pedrosa et al., 2011; Tirapelle et al., 2013). Nevertheless, in the Δ*phaP1* mutant, PhaP2 replaced PhaP1 on the surface of the granules (Tirapelle et al., 2013). To see if this functional complementarity was also reflected in conservation of function, we deleted *phaP1* or *phaP2* or both genes to analyze the effect of phasin absence on PHB accumulation in the mutants. At the same time, we determined the expression patterns of the genes in transcriptional fusions to a *lacZ* reporter-gene housed in different genetic backgrounds, to address if *phaP2* expression enhances upon deletion of *phaP1*, as related previously for other examples of redundant genes (Bratlie et al., 2010; Kondrashov, 2012).

## Materials and Methods

### Bacterial Strains, Plasmids, and Growth Conditions

Strains and plasmids used in this work are listed in **Table 1**. *Escherichia coli* strain Top10 (Thermo Fisher Scientific Inc., Waltham, MA, USA) and S17.1 (Simon et al., 1983) were used for cloning and conjugation procedures, respectively, while strain ET8000 (MacNeil et al., 1982) was used in expression assays with *lacZ* fusions. *E. coli* strains were grown at 37°C in LB medium and shaken at 160 rpm. *H. seropedicae* parental strain SmR1 (Souza et al., 2000) and mutant strains were grown in NFbHP media with 37 mM _DL_-malate or 25 mM glucose and 20 mM NH_4_Cl at 30°C and shaken at 120 rpm (Pedrosa and Yates, 1984).

**Table 1:** Bacterial strains and plasmids used in this work.

Strain or plasmid	Relevant characteristics	Reference
***Escherichia coli* strains**		
Top10	Cloning strain	Invitrogen
S17-1	Conjugation strain	Simon et al., 1983
ET8000	Wild-type strain	MacNeil et al., 1982
***Herbaspirillum seropedicae* strains**		
SmR1	Parental strain, Nif^+^, Sm^R^	Souza et al., 2000
Δ*phaP1*	Chromosomal deletion of *phaP1*	Tirapelle et al., 2013
Δ*phaP2*	Chromosomal deletion of *phaP2*	This work
Δ*phaP1.2*	Chromosomal deletion of *phaP1* and *phaP2*	Tirapelle et al., 2013
Δ*phaC*1	Chromosomal deletion of *phaC1*	Tirapelle et al., 2013
**Plasmids**		
pTZ18R	Cloning plasmid	Mead et al., 1986
pTZ57R/T	T/A cloning plasmid	Fermentas
pDK6	Expression vector *tac* promoter *lacI*^q^, Km^R^	Kleiner et al., 1988
pMMS31	Derivative of pDK6 encoding PhbF from *H. seropedicae* SmR1	Kadowaki et al., 2011
pMP220	RK2 derivative, low-copy number, promoterless *lacZ* containing vector used to construct transcriptional fusions; Tc^R^	Spaink et al., 1987
pEFT11	pMP220 harboring the 5′-flanking region of *phaP*1 cloned upstream of *lacZ*	This work
pEFT12	pMP220 harboring the 5′-flanking region of *phaP*2 cloned upstream of *lacZ*	This work
pK18*mobsacB*	Suicide vector; Km^R^, *sacB*, mobilizable plasmid	Schafer et al., 1994
pEFT13	Deletion product Δ*phaP*2 cloned into the pK18*mobsacB*	This work
pBBR1MCS3	Broad-host-range vector	Kovach et al., 1995
pLPA01	pBBR1MCS3 harboring *phaP*1 of *H. seropedicae*. Over-expression of PhaP1	This work
pLPA02	pBBR1MCS3 harboring the *phaP*2 of *H. seropedicae*. Over-expression of PhaP2	This work

### Quantification of PHB

PHB was quantified by methanolysis and GC-FID (gas chromatography coupled to flame-ionization detector) analyses as described previously (Braunegg et al., 1978) on 5 to 10 mg of lyophilized bacteria. Amounts of PHB in each sample were normalized to the cell dry weight (cdw; weight of the lyophilized bacterial pellet) and expressed as % of PHB cell dry weight^-1^.

### Construction of Mutants of *H. seropedicae* SmR1

The Δ*phaP1* and Δ*phaC1* mutants were constructed by in-frame deletion of the *phaP1* (Hsero_1639, GenBank: 9402240) and *phaC1* (Hsero_2999, GenBank: 9403600) in *H. seropedicae* SmR1, as previously described (Tirapelle et al., 2013). The in-frame marker-less deletion of *phaP2* (Hsero_4759, GenBank: 9405360) was obtained by cloning upstream and downstream fragments of the gene into the non-replicating plasmid pK18*mobsacB*, which carries a kanamycin resistance cassette along with *sacB* that confers sucrose sensitivity (Schafer et al., 1994). Briefly, 500 bp fragments to either flank of *phaP2* were amplified by PCR with primers Fw_*phaP2*_UP and Rev_*phaP2_*UP (sequences showed in Supplementary Table S1) for the upstream region whereas Fw_*phaP2_*DOWN and Rev_*phaP2*_DOWN were used for the downstream region. The PCR products were cloned into pTZ57R/T and sequenced using universal and reverse M13 primers. The downstream fragment was ligated to the upstream fragment using the KpnI site, resulting in the deletion product Δ*phaP2*. The entire construction was digested with BamHI and SalI, in order to ligate the Δ*phaP2* fragment into pK18*mobsacB* digested with the same enzymes, yielding pEFT13. *E. coli* S17-1 was transformed with pEFT13 and the plasmid conjugated to *H. seropedicae* SmR1 using bi-parental mating. Single-recombinants were selected on NFb-malate agar containing streptomycin 80 μg mL^-1^, nalidixic acid 5 μg mL^-1^ and kanamycin 500 μg mL^-1^. A single-recombinant colony was collected in 3 mL NFb-malate and cultivated overnight without antibiotics. The culture was serially diluted and plated on NFb-malate agar containing 10% (w/v) sucrose. Colonies that grew on sucrose were screened for deletion by PCR using the primers Fw_*phaP2*_UP and Rev_*phaP2*_DOWN. To obtain the double mutant Δ*phaP1.2*, the plasmid pEFT13 was conjugated in the Δ*phaP1* mutant of *H. seropedicae* and double-recombinants were selected as described above.

### Construction of Transcriptional Fusions

The intergenic regions of *phaP1* (333 bp including 28 bp of the *phaP1* coding sequence) and *phaP2* (224 bp including 54 bp of the *phaP*2 coding sequence) were amplified from *H. seropedicae* SmR1 genomic DNA by PCR (primer sequences showed in Supplementary Table S1) and cloned into pMP220 (Spaink et al., 1987) upstream of a promoterless, rbs-containing *lacZ* to yield plasmids pEFT11 and pEFT12, respectively.

### RNA Extraction and RT-PCR

Strains were grown on NFb-malate medium with 20 mM of ammonium chloride at 30°C and shaken at 120 rpm. Cells from 1.5 mL of culture at OD_600_ of 1.0 were collected by centrifugation (10,000 × *g*, 4°C, 5 min) and re-suspended in 1 mL of TRIzol^®^ Reagent (Life Technologies, USA). The homogenized sample was incubated for 5 min at room temperature and then, extracted with 0.25 mL of chloroform. After centrifugation (10,000 × *g*, 4°C, 5 min), the aqueous phase was precipitated with 0.5 mL of isopropanol. The pellet was collected by centrifugation (10,000 × *g*, 4°C, 5 min) and washed with 1 mL of ethanol 80%. The air dried RNA pellet was resuspended in 30 μL of RNAse-free water and the quality of RNA preparation was determined by *A*_260_/*A*_280_ ratio and checked by electrophoresis in 1.0% agarose gel. For cDNA preparation, 100 ng of total RNA was used in 20 μL reactions applying the High Capacity RNA-to-cDNA kit (Applied Biosystems, USA) according to the manufacturer’s instructions. From the cDNA preparations, 1 μL was used as template in a 20 μL PCR reaction with PfuX7 as described previously (Norholm, 2010), applying the specific primers described in Supplementary Table S1, 60°C as annealing temperature and 30 cycles of reaction. From each reaction, 2 μL were applied on a 1.5% agarose gel to visualize the intensity of the bands after ethidium bromide staining. The *rrsA* gene encoding the 16S rRNA was used as endogenous control. Negative controls were performed using as template samples of RNA untreated with reverse transcriptase.

### Complementation of *phaP* Mutants

*phaP*1 (Hsero_1639) plus 333 bp of the intergenic region upstream of its start codon was amplified by PCR with Fw_Pro_*phaP1* and Rev_Gen_*phaP1* primers (Supplementary Table S1) from *H. seropedicae* SmR1 genomic DNA and cloned into the XhoI and XbaI sites of pBBR1MCS-3 (Kovach et al., 1995), generating pLPA01. Similarly, *phaP*2 (Hsero_4759) plus 224 bp of the intergenic region upstream of its start codon was amplified with Fw_Pro_*phaP2* and Rev_Gen_*phaP2* primers (Supplementary Table S1) by PCR and cloned into the XhoI and XbaI sites of the pBBR1MCS-3, generating pLPA02. Conjugation was performed by bi-parental mating between *H. seropedicae* SmR1 and *E. coli* S17-1.

### β-Galactosidase Activity Assay

β-galactosidase activity was determined in *E. coli* ET8000 (grown in LB media) carrying the transcriptional fusion plasmids pEFT11 or pEFT12 in the presence of pMMS31 (that expresses the PhaR from *H. seropedicae*) or pDK6 (negative control). To perform similar analyses in *H. seropedicae* parental strain SmR1 and mutant strains, pEFT11 or pEFT12 was introduced by bi-parental mating with *E. coli* S17-1. Transconjugants were selected on NFb-malate agar containing 20 mM of ammonium chloride and tetracycline (10 μg.mL^-1^). β-galactosidase activity was assayed following previous protocol (Miller, 1972).

### Fluorescence and Transmission Electron Microscopy

To visualize PHB granules in SmR1 and its *phaP* mutants, fluorescence microscopy was performed after staining with the fluorescent probe Nile Red, which stains neutral lipids (Spiekermann et al., 1999). Bacterial cultures (1 mL) were harvested by centrifugation for 60 s at 10,000 × *g*. The pellets were resuspended in 30% (v/v) ethanol in PBS (Na_2_HPO_4_ 10 mM, KH_2_PO_4_ 1.8 mM, NaCl 130 mM) and 3 μL of 1.6 mM Nile Red (λ_ex_ 586–579 nm; λ_em_ 637–597 nm) dissolved in DMSO was added and incubated in the dark for 5 min. Then, the samples were centrifuged again for 60 s at 10,000 × *g*, resuspended in PBS and viewed under a fluorescent microscope. The images were obtained using an Axio Imager Z2 microscope (Carl Zeiss Microscopy GmbH, Jena, Germany), equipped with four Metafer automated capture software (Metasystems GmbH, Altlussheim, Germany) and a CoolCube 1 camera (with 100× magnification). To TEM analyses, cell pellets from *H. seropedicae* cultures were fixed with Karnovsky’s fixative (Karnovsky, 1965), post-fixed with 2% OsO_4_ in 0.1 M cacodylic acid buffer (pH 7.2) for 1 h and embedded in Epon 812 (Luft, 1961). After contrasting with 2% uranyl acetate (Watson, 1958) and lead citrate (Reynolds, 1963), samples were examined with a JEOL-JEM 1200 EX II transmission electron microscope.

### Phylogenetic Analysis of Phasin Sequences

The amino acid sequences used in the analysis are listed in the Supplementary Figure S1. The alignment was performed by Muscle (Edgar, 2004) into the MEGA software package (Tamura et al., 2013), using default parameters. The resulting alignment was cured by Gblocks to remove poorly aligned positions and highly divergent regions (Castresana, 2000). The phylogeny reconstruction of the sequences in the cured alignment was obtained by the Neighbor-joining method and tested by bootstrap (10,000 replicates).

### Statistical Analysis

Where appropriate, statistical analysis was carried out using independent two-sample *t*-test with the R package (R Development Core Team, 2015).

## Results

### *H. seropedicae* SmR1 Contains Two Paralogous Phasin and a Third Less Conserved Putative Phasin

**FIGURE 1:**
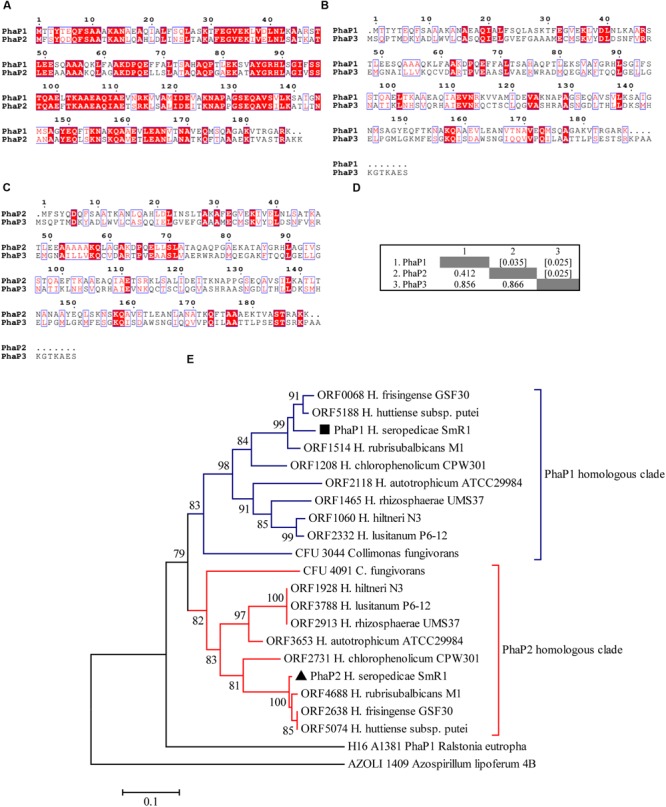
**Protein sequence alignments of phasins from *Herbaspirillum seropedicae* SmR1 and phylogenetic analysis of homologous sequences.** Multiple alignments of protein sequences of **(A)** PhaP1 (Hsero_1639) and PhaP2 (Hsero_4759), **(B)** PhaP1 and PhaP3 (Hsero_2402), and **(C)** PhaP2 and PhaP3 were carried out using Espript 3.0 and the default parameters (Robert and Gouet, 2014). The red shaded letters indicate identical residues, while red letters represent conserved residues. **(D)** The number of amino acid differences per site from between PhaP1, PhaP2, and PhaP3 sequences are shown. Standard error estimate(s) are shown above the diagonal (enclosed within brackets) and were obtained by a bootstrap procedure (10000 replicates). All ambiguous positions were removed for each sequence pair. There were a total of 199 positions in the final dataset. Evolutionary analyses were conducted in MEGA6 (Tamura et al., 2013). **(E)** The evolutionary history was inferred using the Neighbor-Joining method (Saitou and Nei, 1987). The putative phasin of *Azospirillum lipoferum* encoded by the AZOLI 1409 (Wisniewski-Dye et al., 2011) was applied as an outgroup. Only nodes with bootstrap test (10000 replicates) bigger than 70% are shown next to the branches (Efron et al., 1996). The tree is drawn to scale, with branch lengths in the same units as those of the evolutionary distances used to infer the phylogenetic tree. The evolutionary distances were computed using the *p*-distance method and are in the units of the number of amino acid differences per site. Evolutionary analyses were conducted in MEGA6 (Tamura et al., 2013). The PhaP sequences used to construct the tree are shown in Supplementary Figure S1.

Three genes coding putative phasins (*phaP1*, locus-tag Hsero_1639; *phaP2*, Hsero_4759 and *phaP3*, Hsero_2402; Kadowaki et al., 2011; Pedrosa et al., 2011) are present in *H. seropedicae* SmR1. The alignments and pair-wise distances between PhaP1 and PhaP2 showed a short evolutionary distance (0.412 using the *p*-distance method), but PhaP3 was further removed, indicating significant sequence divergence and possibly also function (**Figures 1A–D**). Indeed, PhaP3 was only detected on PHB granules when *phaP1* was deleted and, it was clearly less abundant than PhaP2, the main phasin in the absence of PhaP1. The *phaP1* and *phaP2* genes could have been generated by gene duplication based on 59% identity between their encoded amino acid sequences, as well as a region of more than 150 amino acids that can be aligned (**Figure 1A**), according to the classification of duplicated genes proposed by Gevers et al. (2004). Nevertheless, the horizontal gene transfer (HGT) of an additional phasin gene cannot be totally ruled out, as intra-genome homologs are also acquired via HGT (Maerk et al., 2014). Accordingly, the co-occurrence of *phaP1* and *phaP2* homologs was found in other bacteria phylogenetically close to *H. seropedicae* SmR1 (**Figure 1E**). The phylogenetic analysis of PhaP1 and PhaP2 homolog sequences showed that the appearance of a second phasin occurred early in evolution of the *Herbaspirillum* genus and was conserved in subsequent speciation events (**Figure 1E**), indicating that the presence of both phasins should be important for PHB accumulation.

### Deletion of *phaP1* Reduces PHB Accumulation

**FIGURE 2:**
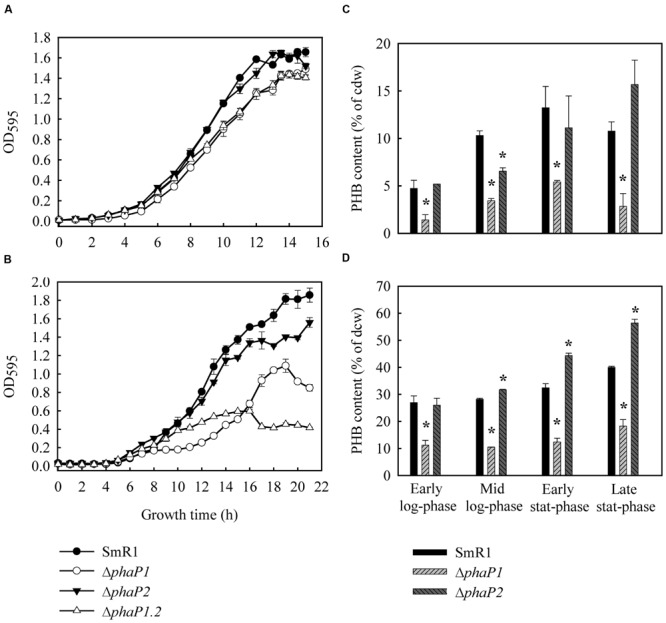
**Growth and PHB accumulation profiles of *H. seropedicae* SmR1 (parental strain) and the mutants Δ*phaP1*, Δ*phaP2*, and Δ*phaP1.2*.** Strains were grown in NFb medium with 20 mM of ammonium chloride and 37 mM _DL_-malate **(A,B)** or 25 mM (w/v) D-glucose **(C,D)** at 30°C (orbital agitation at 120 rpm). OD_595_ growth data were obtained from three independent cultures, while PHB contents were determined on four independent samples. PHB levels in Δ*phaP1.2* were less than 1% at all growth phases. Where appropriate, statistical significance is shown (^∗^*p*-value ≤ 0.05, independent two-sample *t*-test). The data obtained to SmR1 was used for normalization and determination of statistical significance.

To verify the roles of PhaP1 and PhaP2 in PHB accumulation, three isogenic deletion mutants – Δ*phaP1*, Δ*phaP2*, and Δ*phaP1.2* – were constructed. The mutant strains grew equally well as the parental strain on NFb-malate medium (**Figure 2A**; **Table 2**). Since cells accumulating PHB are less translucent than those without PHB, which can affect optical density measurements, the growth of parental and mutant strains was also determined by counting the number of viable cells in culture (Supplementary Figure S2). Intracellular accumulation of PHB in the strains grown on malate was determined: (i) in early exponential growth (OD_600_ = 0.6); in mid-exponential growth (OD_600_ = 0.8); (iii) early stationary phase (OD_600_ = 1.2); and, (iv) in late stationary growth (OD_600_ = 1.4). The parental strain accumulated PHB up to 13% (w/w) of cell dry weight (cdw) after 12 h of growth (early stationary phase; **Figure 2B**), but decreased to 11% of PHB at late stationary phase. Similar behavior was observed with Δ*phaP2* which reached 15% of cdw at early stationary phase. On the other hand, Δ*phaP1* only accumulated approximately 50% of parental levels of PHB at all tested growth phases (**Figure 2B**). Deletion of both phasins (Δ*phaP1.2*) drastically reduced PHB accumulation to <1% of cdw (**Figure 2B**).

**Table 2:** Growth rates of *phaP* mutants in NFb media containing malate or glucose as carbon source.

Strain	Growth rate (ΔOD_595_/h)^a^	
	Malate^b^	Glucose
SmR1 (wt)	0.237 ± 0.007	0.184 ± 0.026^c^
Δ*phaP1*	0.182 ± 0.029	0.123 ± 0.022^d^
Δ*phaP2*	0.200 ± 0.018	0.151 ± 0.017^c^
Δ*phaP1.2*	0.164 ± 0.019	0.055 ± 0.009^e∗^

*^a^The values are averages ± standard errors for two independent experiments. ^b^Growth rate was calculated with the data of OD_600_ between 8 and 13 h of growth for all strains. ^c^Growth rate calculated between 10 and 16 h of growth. ^d^Growth rate calculated between 12 and 19 h of growth. ^e^Growth rate calculated between 7 and 16 h of growth. Where appropriate, statistical significance is shown (^∗^*p*-value ≤ 0.05). The data obtained to the SmR1 strain was used for normalization and determination of statistical significance.*

To exclude the possibility that it was growth on malate rather than the mutations *per se* that affected PHB accumulation, similar experiments were performed using glucose as the carbon source. Under these conditions, SmR1 also accumulated PHB as previously reported for the strains Z67, Z69, and Z78 of *H. seropedicae* (Catalan et al., 2007) and the parental strain reached a maximum of 40% of cdw in late-stationary phase. The production of PHB by Δ*phaP2* was similar to the parental strain at the early stat-phase and reached 56% of cdw at the late stat-phase. On the other hand, the Δ*phaP1* accumulated only 18% of PHB as its maximum production, corresponding to 50% of the PHB content of the parental strain (**Figure 2C**). In Δ*phaP1.2*, PHB accumulation was not detected when cultivated on glucose. Noteworthy, the Δ*phaP1.2* strain presented a growth penalty when cultivated in glucose (**Table 2**), suggesting that the absence of PHB affects the glucose metabolism in *H. seropedicae*. Despite the growth rate in glucose of the Δ*phaP1* mutant did not present a statistical difference (**Table 2**), its growth pattern was atypical, since it stopped to grow early than parental strain (**Figure 2A**). This finding is also in agreement that reduction in PHB affects growth in glucose. In conclusion, regardless of the carbon source employed and the growth phase, the absence of PhaP1 or both phasins negatively affected PHB accumulation.

### Microscopic Alterations in the Number of Granules Per Cell

**FIGURE 3:**
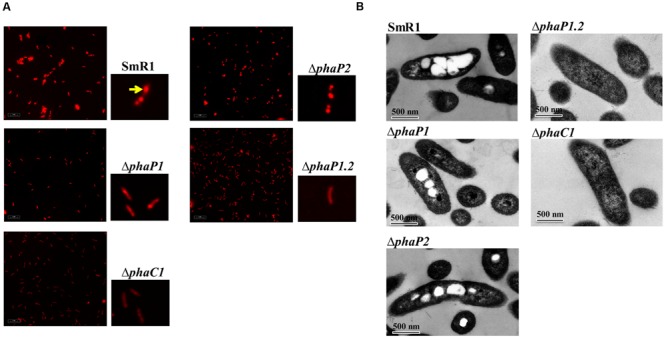
**Fluorescence microscopy and transmission electron microscopy of *H. seropedicae* SmR1, *phaP* and *phaC1* mutants. (A)** Strains were grown in NFb medium with 37 mM _DL_-malate and 20 mM NH_4_Cl to an OD_600_ of 1.0. Cells were stained with Nile Red and visualized by excitation with 543 nm light. The yellow arrow in the SmR1 panel indicates a typical PHB granule stained with Nile Red. **(B)** The strains cultivated in NFb medium with 37 mM _DL_-malate and 20 mM NH_4_Cl to an OD_600_ of 1.0 were processed and visualized by TEM. The black bar represents 500 nm in scale.

After staining with Nile Red, 92% of native cells contained at least two PHB granules per cell (**Figure 3**) the rest only one (*n* = 100 counted cells), but in Δ*phaP1* the situation was practically reversed – only 18% of cells contained two granules and 82% only one (*n* = 100 cells). Δ*phaP2* had a similar granular distribution as the parental strain. No cells containing two PHB granules in Δ*phaP1.2* were observed, but 9% of the counted cells (*n* = 100) contained one PHB granule. In the strict sense, it was not clear whether these PHB granules were coated by other proteins or a transient agglomeration of polymer within the cytoplasm, since at all times examined, the level of PHB accumulated in the double mutant was below 1% of cdw. The absence of granules in Δ*phaP1.2* was also confirmed by TEM analysis (**Figure 3B**).

### Expression of *phaP1* and *phaP2* Is Repressed by PhaR

Previously, we showed that the negative regulator PhaR of *H. seropedicae* (a homolog of PhaR in *R. eutropha* H16) binds to the regulatory region of *phaP1* thus repressing its expression (Maehara et al., 2001, 2002; Pötter et al., 2002; York et al., 2002; Kadowaki et al., 2011). To further examine the role of PhaR on expression of *phaP1* and *phaP2*, we fused a promoterless rbs-containing *lacZ* downstream of the regulatory regions of both genes (hereafter denoted P*phaP1*-*lacZ* and P*phaP2*-*lacZ*). We decide to measure the activity of the fusions in *E. coli* as a heterologous non-PHB-producing model, to avoid any perturbation on PhaR activity caused by PHB production (Maehara et al., 2002). The fusions were transformed into *E. coli* ET8000 expressing or not *H. seropedicae* PhaR. Cells carrying P*phaP1*-*lacZ* and P*phaP2*-*lacZ* fusions showed similar, high β-galactosidase activities when PhaR was not expressed. However, cells expressing PhaR had a remarkable repression on expression levels of both P*phaP1-* and P*phaP2-lacZ* (**Table 3**).

**Table 3:** Transcriptional analysis of P*phaP1*-*lacZ* and P*phaP2*-*lacZ* fusions in *E. coli* ET8000 expressing PhaR from *H. seropedicae* SmR1.

Strain	β-galactosidase activity before induction (Miller units)	β-galactosidase activity 2 h after induction (Miller units)
ET8000/pDK6/P*phaP1*-*lacZ*	8,516.5 ± 693.3	11,801.3 ± 642.1
ET8000/pMMS31/P*phaP1*-*lacZ*^∗^	168.0 ± 6.4^∗∗^	89.7 ± 2.5^∗∗^
ET8000/pDK6/P*phaP2*-*lacZ*	11,180.8 ± 142.8	12,061.1 ± 55.5
ET8000/pMMS31/P*phaP2*-*lacZ*^∗^	149.8 ± 3.2^∗∗^	85.5 ± 2.8^∗∗^

*The cultures were grown in LB medium until OD600 of 0.6. At this point, the IPTG was added to final concentration of 1 mM. The activity was monitored for two more hours after inducer addition. ^∗^Strains expressing PhaR from *H. seropedicae* under control of the P*tac* promoter. Where appropriate, statistical significance is shown (^∗∗^*p*-value ≤ 0.01, independent two-sample *t*-test). The data obtained to the strains harboring P*phaP1*- or P*phaP2*-*lacZ* not expressing PhaR were used for normalization and determination of statistical significance.*

### The *phaP1* and *phaP2* Genes Are Dissimilarly Expressed

**FIGURE 4:**
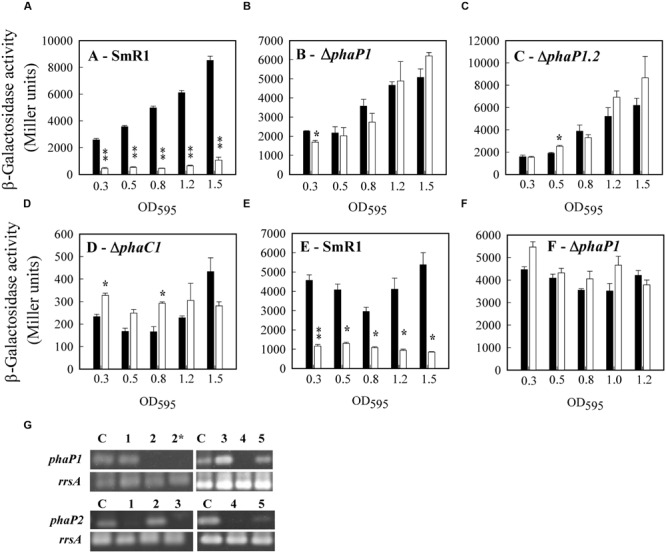
**Transcriptional analyses of *phaP1* and *phaP2* expression in *H. seropedicae*. (A–D)** Cells harboring P*phaP*1-*lacZ* (black bars) or P*phaP2*-*lacZ* (white bars) were grown in liquid NFb medium containing 37 mM _DL_-malate and 20 mM NH_4_Cl at 30°C. **(E,F)** Cells harboring P*phaP*1-*lacZ* (black bars) or P*phaP*2-*lacZ* (white bars) were grown in liquid cultures of NFb containing 25 mM D-glucose and 20 mM NH_4_Cl at 30°C. When the OD_600_ of the cultures reached the indicated values, 100 μL of culture was removed to determine β-galactosidase activity. *H. seropedicae* strains harboring pMP220 (promoterless *lacZ* plasmid) gave an average activity of 50 Miller units in all growth phases analyzed. Experiments were performed in biological triplicates. The means of β-galactosidase activity were tested in pairs for each sample point. Where appropriate, statistical significance is shown (^∗^*p*-value ≤ 0.05, ^∗∗^*p*-value ≤ 0.01, independent two-sample *t*-test). **(G)** RT-PCR analysis. The RNA from the strains SmR1 (lane 1), Δ*phaP1* (lane 2 and 2^∗^ for *phaP1*, lane 2 for *phaP2*), Δ*phaP2* (lane 3), Δ*phaP1.2* (lane 4), and Δ*phaC1* (lane 5) was purified and submitted to direct RT-PCR amplification of *phaP1* and *phaP2* gene as described in Methods. The lane C is a PCR product amplified from gDNA from *H. seropedicae* SmR1 used as positive control of the reaction. The 16S rRNA (*rrsA*) was used as an endogenous expression control. A representative gel from tree independent RNA extractions is showed.

To determine expression levels of *phaP1* and *phaP2*, the P*phaP1*- and P*phaP2*-*lacZ* fusions were conjugated into the parental and mutant strains. Expression profiles were evaluated during growth in media containing _DL_-malate as the carbon source. Expression of P*phaP1*-*lacZ* in the parental strain was dependent on the growth stage, since expression increased after an OD_600_ of 0.5 was achieved and PHB began to accumulate (**Figure 4A**). Interestingly, expression of P*phaP2*-*lacZ* was 8-fold lower than that achieved by the P*phaP1*-*lacZ* fusion in the same strain (**Figure 4A**). However, expression of P*phaP2*-*lacZ* increased 6-fold in Δ*phaP1*, showing that upon deletion of the main phasin, PhaP2 can act as a backup phasin (**Figure 4B**). Since the Δ*phaP1.2* did not accumulate PHB granules, one should anticipate that both fusions would be repressed by PhaR. Nevertheless, the P*phaP1*- and P*phaP2*-*lacZ* fusions were actives in Δ*phaP1.2*, indicating that not only PHB granules de-repress expression of *phaP*, but that newly synthesized chains of PHB have the same effect (**Figure 4C**). As expected, in Δ*phaC1*, which is unable to synthesize PHB, expression of both fusions was repressed (**Figure 4D**). Expression of *phaP1* and *phaP2* was also assayed in NFb media containing 25 mM glucose and 20 mM ammonium chloride, conditions which stimulated PHB accumulation. High β-galactosidase activities (4,000 Miller units) for P*phaP1*-*lacZ* were obtained in all strains analyzed (parental and the mutants), showing that even at low cell densities (OD_600_ ≥ 0.3, **Figure 4E**) enough PHB granules were present to almost completely de-repress *phaP1* expression. Expression of *phaP2* was not fully activated in the parental strain (**Figure 4E**), but in Δ*phaP1* it was fivefold higher even at low cell densities (**Figure 4F**). The results of expression applying *lacZ* fusions were validated by RT-PCR. The same profile of transcription was observed when the intensities of the amplified bands from *phaP1* and *phaP2* cDNAs were compared in different genetic backgrounds (**Figure 4G**).

### Complementation with *phaP1* Fully Restores PHB Accumulation, while *phaP2* Expression Has a Partial Effect

**FIGURE 5:**
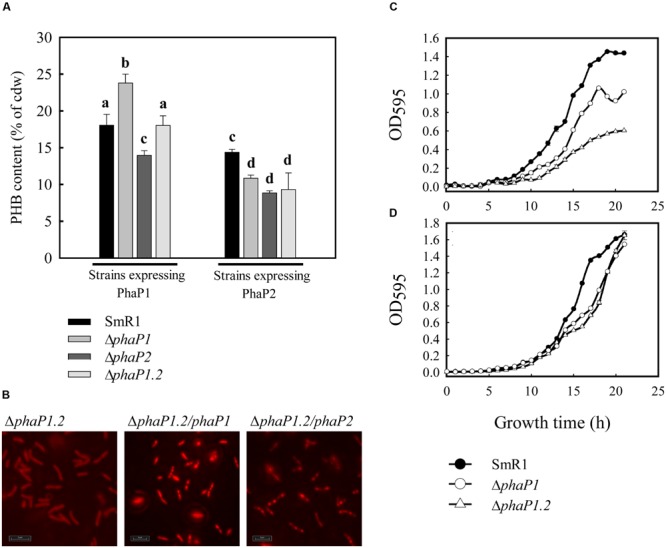
**Poly-3-hydroxybutyrate contents of *phaP* mutants complemented with plasmids harboring *phaP1* and *phaP2*. (A)** *H. seropedicae* strains conjugated with pLPA01 were complemented with *phaP*1 *in trans* under control of its native promoter. The strains conjugated with pLPA02 were complemented with *phaP*2 *in trans* under control of its native promoter. Cells harboring pLPA01 or pLPA02 were grown in liquid NFb containing 37 mM _DL_-malate and 20 mM NH_4_Cl at 30°C. When the OD_600_ of the cultures reached 1.0, 10 mL was removed to determine the PHB content by gas chromatography as described in section “Materials and Methods.” The bars marked with different letters indicate means significantly different (independent two-sample *t*-test, *p* < 0.05). Experiments were performed in quadruplicate. **(B)** Cells of Δ*phaP1.2* mutant complemented with pLPA01 were stained with Nile Red and visualized by excitation with 543 nm light. Strains harboring pBBR1MCS-3 **(C)** or pLPA01 **(D)** were grown in NFb medium with 20 mM of ammonium chloride and 25 mM (w/v) _D_-glucose at 30°C (orbital agitation at 120 rpm). OD_595_ growth data were obtained from three independent cultures.

To determine if both phasins were able to restore PHB accumulation, the *phaP* genes were cloned under control of their native promoter into a medium copy-number plasmid (pBBR1MCS-3) and expressed into the *phaP* mutants. The expression of *phaP1* in the Δ*phaP1*, Δ*phaP2*, and Δ*phaP1*.*2* mutants restored PHB accumulation to native levels (**Figure 5A**). The expression of a plasmid-borne *phaP1* copy restored PHB granules formation in Δ*phaP1*.*2* (**Figure 5B**). Complementation by expression of *phaP2* significantly reduced PHB accumulation, regardless the strain tested. Δ*phaP1* complemented with *phaP2* presented 45% of reduction in PHB as compared to complementation with *phaP1*. Similarly, 51% less PHB was observed when Δ*phaP1.2* was complemented with *phaP2*. Once again, these results show that PhaP1 is more effective in controlling PHB accumulation than its homolog PhaP2. As demonstrated before in this work, deletion of *phaP1* or both phasin genes generated mutant strains with growth penalty in minimal medium containing glucose as sole carbon source (**Figure 1C**). Therefore, to verify if complementation would restore normal growth in NFb-glucose, the strains were complemented with *phaP1* and their growth curves were determined (**Figures 5C,D**). The strains Δ*phaP1* and Δ*phaP1*.*2*, which exhibited reduced growth on glucose, grew as the parental strain when complemented with *phaP1*. In conclusion, the expression of *phaP1* recovered PHB accumulation in the deficient strains, consequently normalizing their metabolic status and turning them able to grow in glucose.

## Discussion

To analyze the role of phasins in *H. seropedicae*, we correlated their presence with PHB production and expression of *phaP1* and *phaP2*. Deletion of *phaP1* reduced PHB accumulation on 50%, showing that PhaP1 is the key phasin controlling synthesis and stability of PHB granules. Deletion of *phaP2* had little effect, reinforcing the idea that when PhaP1 is expressed, PHB granules are well formed and stocked (**Figures 2** and **3**). The suggestion that *H. seropedicae* has other phasin-like proteins seems unlikely, since deletion of both genes completely suppressed PHB granules formation. A similar dependence of phasins on accumulation of PHB was also found in *S. meliloti* Rm1021 (Wang et al., 2007), in which deletion of *phaP1*, the main phasin in this bacterium, reduced PHB accumulation by 20% and increased the doubling time by 2.3 h (Wang et al., 2007). Similarly, deletion of *phaP2* did not significantly affect growth and PHB production of *S. meliloti*, however, deletion of both phasins increased the doubling time by 3.6 h and fully abrogated PHB accumulation (Wang et al., 2007). In the insect gut symbiont *Burkholderia* sp. RPE75, the *phaP* deletion reduced PHB accumulation by 2.8-fold and the distribution of PHB granules was heterogeneous among the cells of the Δ*phaP* mutant (Kim et al., 2013). A total of six *phaP* genes were identified within the genome of *Burkholderia* sp. RPE75, therefore it was suggested that the redundancy among phasins could maintain PHB production in the Δ*phaP* mutant and render it with less impact on bacterial-insect symbiosis (Kim et al., 2013). In fact, from our results it is likely that the expression of a backup phasin ensures some level of PHB accumulation and, hence, reduces the impact on bacterial fitness. Possibly, the same mechanism might be occurring in the Δ*phaP* strain of *Burkholderia* sp. RPE75. Recently, Hauf et al. (2015) reported the deletion of the *ssl2501* expressing a phasin in *Synechocystis* sp. PCC 6803. The Δ*ssl2501* mutant presented a reduction in the number of PHB granules per cell and an increase in the mean PHB granule size, however, the PHB content was only slightly lower in the mutant. Once again, as the deletion of *ssl2501* alone has not abolished PHB accumulation, it was also suggested that possibly in *Synechocystis*, other phasin-like proteins might be expressed (Hauf et al., 2015).

These data contrast with the situation in the well-studied model *R. eutropha* H16, in which deletion of the four phasin genes did not completely impair PHB accumulation (Kuchta et al., 2007). Besides these four phasins, three other proteins have been recently reported in *R. eutropha* H16, namely PhaP5, PhaP6, and PhaP7 (Pfeiffer and Jendrossek, 2011, 2012). The deletion of these additional phasin genes alone did not affect PHB granules formation and the content accumulated in *R. eutropha* (Pfeiffer and Jendrossek, 2011, 2012). To the best of our knowledge, a mutant of *R. eutropha* with all phasin genes deleted (*phaP1* to *phaP7*) has not been constructed so far. This mutant could reveal whether all phasins are relevant to PHB granule biogenesis and accumulation in *R. eutropha* and, if the functional redundancy among phasins (including PhaP5-7) supports PHB accumulation in the multiple deletion mutants, as related to Δ*phaP1234* strain (Kuchta et al., 2007).

A well accepted model for transcriptional regulation of *phaP* genes in *R. eutropha* H16 and *Paracoccus denitrificans* (Maehara et al., 2001, 2002; Pötter et al., 2002; York et al., 2002; Yamada et al., 2007, 2013) suggested that the transcriptional repressor PhaR binds to the regulatory region upstream of *phaP* genes, blocking gene transcription. At the onset of PHB synthesis, the PhaR repressor is sequestered from DNA by PHB and transcription is initiated. PhaR thus couples PHB synthesis with phasin expression (York et al., 2002). In *H. seropedicae* SmR1, we have shown that PhaR (previously named PhbF) also functions as a repressor of transcription (Kadowaki et al., 2011) and here we have extended these observations to include repression of *phaP1* and *phaP2* expression (in *E. coli*; **Table 3**). Furthermore, the pattern of *phaP1* and *phaP2* expression in different backgrounds of *H. seropedicae* demonstrated a backup regulation, whereas the genes are dissimilarly expressed (**Figure 4**). In other words, the simultaneously expression of both phasins seems to be unnecessary, however, upon mutation of *phaP1*, expression of *phaP2* is reprogrammed to achieve a similar level compared to expression of *phaP1* in the wild type. This mode of regulation is named responsive backup circuit (RBC) and has important consequences controlling expression of functional redundant proteins to increase the robustness of organisms when facing stressful conditions (Kafri et al., 2006). Clearly, our results demonstrated that the expression of *phaP2* in the Δ*phaP1* reduced the negative impact on PHB accumulation and growth in glucose, rendering a more fit phenotype than the Δ*phaP1*.*2* strain.

The existence of two highly homologous genes encoding proteins with the same predicted function raises the question of whether the phasins of *H. seropedicae* are genuinely redundant. It is unlikely that *phaP1* and *phaP2* represent truly redundant genes, as deletion of *phaP1* clearly resulted in a less fit phenotype. Therefore, it is possible that due to its greater efficiency, PhaP1 has been selected as the main phasin and, as consequence, the expression of *phaP2* was attenuated, converting PhaP2 in a backup phasin. This assumption raises two important points: (i) it guarantees proper levels of phasin expression, avoiding perturbations on PHB synthesis and granule formation and (ii) it saves the cells of wasting unnecessary metabolic costs with superfluous gene expression. Other β-Proteobacteria including species of the *Herbaspirillum* genus, *Collimonas fungivorans, Herminiimonas arsenicoxydans* and *Janthinobacterium* sp. possess orthologous to *phaP1* and *phaP2* of *H. seropedicae*, indicating that the backup expression of *phaP2* may be conserved among species phylogenetically related to *H. seropedicae*. In the genome of *Azoarcus* sp. BH72, a plant-associated and PHB-producing β-proteobacterium, four genes expressing putative phasins were found (Krause et al., 2006), suggesting that the backup expression of phasins may be an important mechanism to maintain PHB production also in other species less phylogenetically close to *Herbaspirillum*. Interestingly, in other well characterized plant-associated and PHB-producing bacteria as *A. brasilense* Sp245 and *A. lipoferum* 4B, the genome sequencings revealed only one probable gene encoding a phasin for each organism, AZOBR_p110146 and AZOLI_1409, respectively (Wisniewski-Dye et al., 2011). On the other hand, in *A. brasilense* FP2 (a mutant strain from *A. brasilense* Sp7 resistant to nalidixic acid and streptomycin) were found four phasin genes, which are expressed when the bacteria were epiphytically colonizing roots of *Triticum aestivum* (Camilios-Neto et al., 2014). To the best of our knowledge mutansts defective in phasin expression were not constructed to *Azospirillum* species so far. Therefore, these findings pave the way to investigate the impact of phasin gene deletions in other relevant plant-associated bacteria, as those from *Azoarcus* and *Azospirillum* genus.

## Author Contributions

LA and CT designed and performed the most part of experiments, analyzed the data and wrote the manuscript. ET constructed mutants and some plasmids used in this work. LD performed the TEM analysis. MT-S prepared cDNA and performed the RT-PCR. MS, ES, and FO conceived and supervised the study. LC and MM-S conceived and supervised the study, analyzed the data and wrote the manuscript.

## Conflict of Interest Statement

The authors declare that the research was conducted in the absence of any commercial or financial relationships that could be construed as a potential conflict of interest.
