# Supplementary material for: Backup Expression of the PhaP2 Phasin Compensates for phaP1 Deletion in Herbaspirillum seropedicae, Maintaining Fitness and PHB Accumulation
Source: Front Microbiol. 2016 May 20;7:739. doi: 10.3389/fmicb.2016.00739 (PMC4873508; doi:10.3389/fmicb.2016.00739)
Supplement: Supplementary file 1 [file Table_1.DOC]

Supplementary Material

Backup expression of the PhaP2 phasin compensates for *phaP1* deletion in *Herbaspirillum seropedicae*, maintaining fitness and PHB accumulation

**Luis Paulo Silveira Alves^1^, Cícero Silvano Teixeira^1^, Evandro Freire Tirapelle^1^, Michelle Zibetti Tadra-Sfeir^1^, Maria Berenice Reynaud Steffens^1^, Emanuel Maltempi de Souza^1^, Fabio de Oliveira Pedrosa^1^, Leda Satie Chubatsu^1^, Marcelo Müller-Santos^1*^**

*** Correspondence:** Marcelo Müller-Santos: [marcelomuller@ufpr.br](mailto:marcelomuller@ufpr.br)

**Supplementary Table 1.** Primers used in this work

| Primer | Sequence (5’ → 3’) | Restriction site | Purpose |
| --- | --- | --- | --- |
| Fw_*phaP2*_UP | GGATCCCGCAAAGCCAG | BamHI | *phaP2* upstream region amplification |
| Rev_*phaP2_*UP | GGTACCGGCGGAAAACT | KpnI |  |
| Fw_*phaP2*_DOWN | GGTACCTTCACCGCTGC | KpnI | *phaP2* downstream region amplification |
| Rev_*phaP2_*DOWN | GTCGACGAACACCAAGGG | SalI |  |
| Fw_prom_*phaP1* | AGATCTCACACCACACTCTCG | BglII | Amplification of *phaP1* upstream region (transcriptional fusion with *lacZ*) |
| Rev_prom_*phaP1* | CTGCAGCGGAAAATTGCTC | PstI |  |
| Fw_prom_*phaP2* | GTTAGATCTTCGTGGTCCATGACCTGCCC | BglII | Amplification of *phaP2* upstream region (transcriptional fusion with *lacZ*) |
| Rev_prom_*phaP2* | ATCCTGCAGTCTTGGTACGAGAACATGGTC | PstI |  |
| Fw_*phaP1*_RT | TACACCGAGCAATTTTCCGC |  | *phaP1* RT-PCR |
| Rev_*phaP1*_RT | GCTGGTCAGTGCGAAGAATT |  |  |
| Fw_*phaP2*_RT | CCAGGCTGAATTCACCAAGG |  | *phaP2* RT-PCR |
| Rev_*phaP2*_RT | GGCTTGCTTGGAGTTCTTCG |  |  |
| Fw_*rrsA*_RT | TGGTAGTCCACGCCCTAAAC |  | *rrsA* RT-PCR |
| Rev_*rrsA*_RT | TCGAGCACTCCCAAATCTCT |  |  |
| Fw_Pro_*phaP1* | CTCGAGTGCAACAAAACTGCC | XhoI | *phaP1* cloning for complementation |
| Rev_Gen_*phaP1* | TCTAGAAGCGCCTGAAACGGC | XbaI |  |
| Fw_Pro_*phaP2* | CTCGAGCGGGTAAAAGAACCG | XhoI | *phaP2* cloning for complementation |
| Rev_Gen_*phaP2* | TCTAGACGCAGTCCTGATGGC | XbaI |  |
