# Supplementary material for: Backup Expression of the PhaP2 Phasin Compensates for phaP1 Deletion in Herbaspirillum seropedicae, Maintaining Fitness and PHB Accumulation
Source: Front Microbiol. 2016 May 20;7:739. doi: 10.3389/fmicb.2016.00739 (PMC4873508; doi:10.3389/fmicb.2016.00739)
Supplement: Supplementary file 2 [file Image_1.PDF]

Supplementary Figure 1

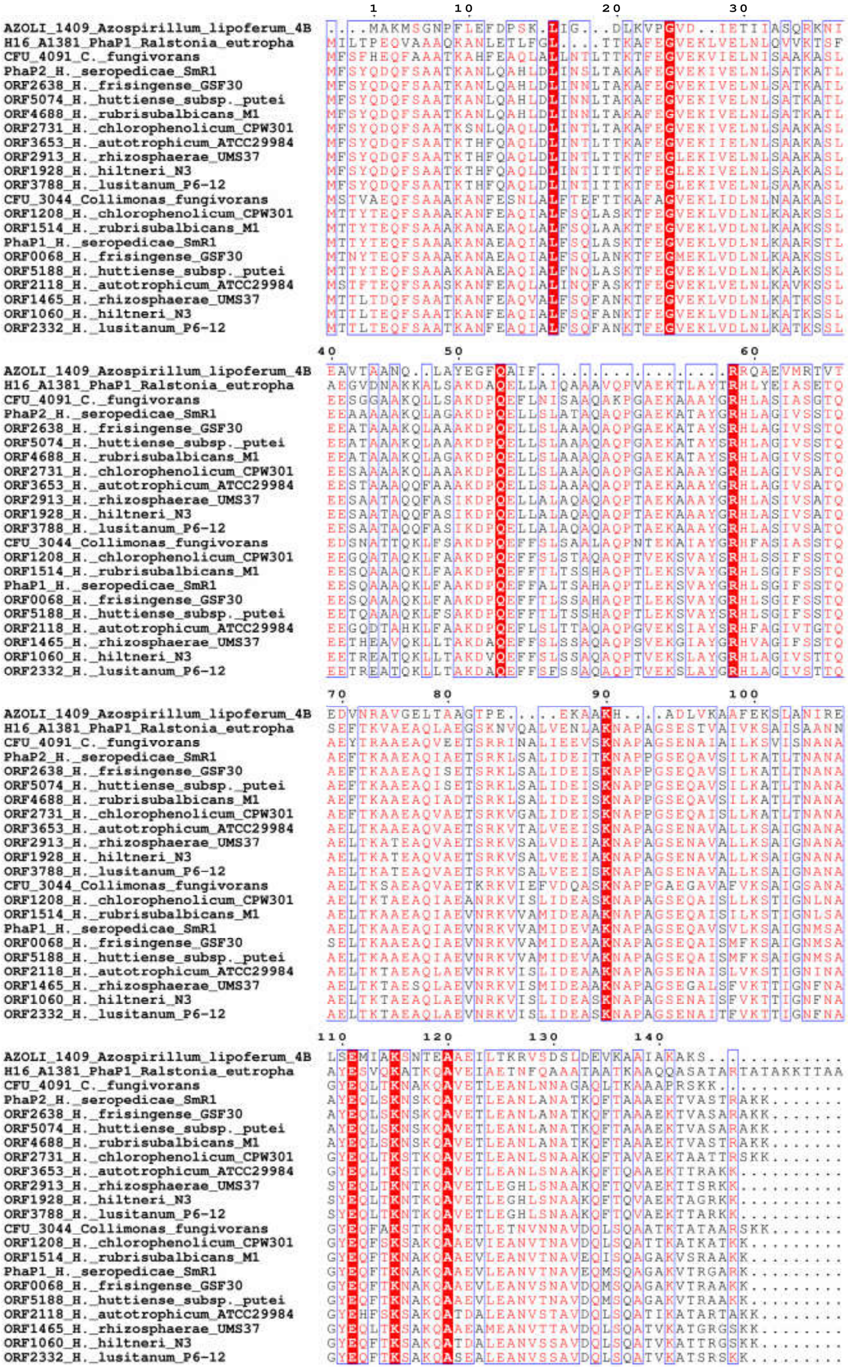

Supplementary Figure 1. Protein sequence alignment of phasins from other species of *Herbaspirillum*, *Collimonas fungivorans*, *Ralstonia eutropha* and *Azospirillum lipoferum* used to the phylogenetic analysis. Multiple alignment of phasins was carried out using Espript 3.0 and the default parameters (Robert and Gouet, 2014). The red shaded letters indicate identical residues, while red letters represent conserved residues. The phasin PhaP from *A. lipoferum* 4B was applied as an outgroup.
