# Supplementary material for: Backup Expression of the PhaP2 Phasin Compensates for phaP1 Deletion in Herbaspirillum seropedicae, Maintaining Fitness and PHB Accumulation
Source: Front Microbiol. 2016 May 20;7:739. doi: 10.3389/fmicb.2016.00739 (PMC4873508; doi:10.3389/fmicb.2016.00739)
Supplement: Supplementary file 3 [file Image_2.PDF]

Supplementary Figure 2

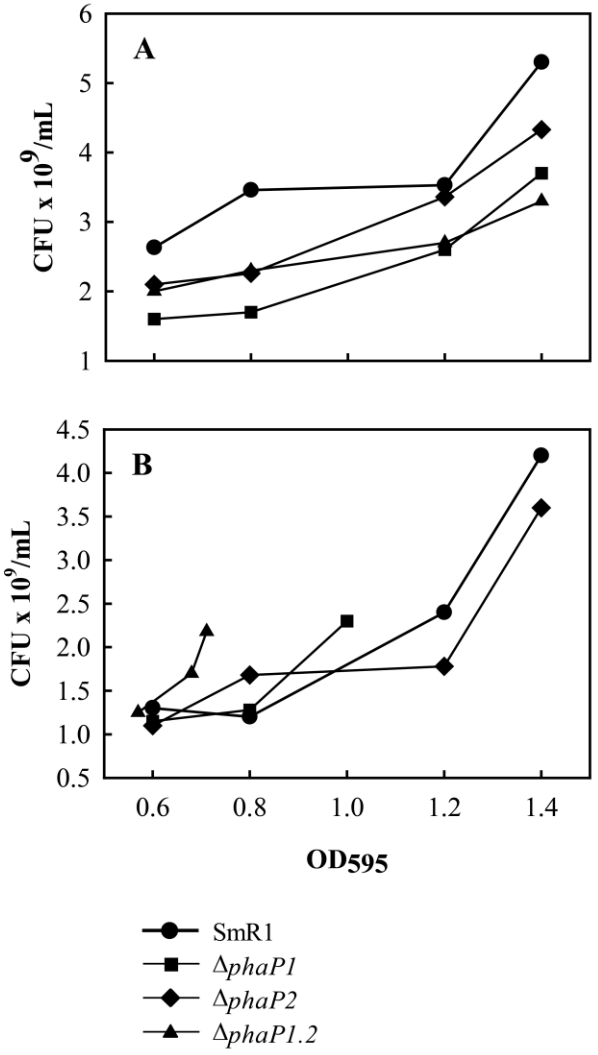

Supplementary Figure 2. Growth of *H. seropedicae* SmR1 (parental strain) and the mutants Δ*phaP1*, Δ*phaP2* and Δ*phaP1.2*. Strains were grown in NFb medium with 20 mM of ammonium chloride and 37 mM DL-malate (**A**) or 25 mM (w/v) D-glucose (B) at 30°C (orbital agitation at 120 rpm). CFU counts were obtained from three independent cultures though serial dilution and plating in NFb-malate agar with 20 mM of ammonium chloride. Due the growth defect of the Δ*phaP1* and Δ*phaP1.2* strains in glucose, their growths were monitored until the OD_{595} of the cultures stopped increasing.
